# Social behavior in RASopathies and idiopathic autism

**DOI:** 10.1186/s11689-021-09414-w

**Published:** 2022-01-12

**Authors:** Allison M. H. Foy, Rebekah L. Hudock, Ryan Shanley, Elizabeth I. Pierpont

**Affiliations:** 1 Department of Pediatrics, Division of Clinical Behavioral Neuroscience, University of Minnesota Medical School, 2025 East River Parkway, Minneapolis, MN 55414 USA; 2 Department of Educational Psychology, University of Wisconsin-Madison, Madison, USA; 3 Biostatistics Core, Masonic Cancer Center, University of Minnesota, Minneapolis, USA

**Keywords:** Prosocial, Social skills, Social function, Social competence, Autism, RASopathy, Costello syndrome, Cardiofaciocutaneous syndrome, Neurofibromatosis type 1, Noonan syndrome

## Abstract

**Background:**

RASopathies are genetic syndromes that result from pathogenic variants in the RAS-MAPK cellular signaling pathway. These syndromes, which include neurofibromatosis type 1, Noonan syndrome, cardiofaciocutaneous syndrome, and Costello syndrome, are associated with a complex array of medical and behavioral health complications. Despite a heightened risk for social challenges and autism spectrum disorder (ASD), few studies have compared different aspects of social behavior across these conditions. It is also unknown whether the underlying neuropsychological characteristics that contribute to social competence and socially empathetic (“prosocial”) behaviors differ in children with RASopathies as compared to children with nonsyndromic (i.e., idiopathic) ASD.

**Methods:**

In this cross-sectional, survey-based investigation, caregivers of preschool and school-aged children with RASopathies (*n* = 202) or with idiopathic ASD (*n* = 109) provided demographic, medical, and developmental information about their child, including psychiatric comorbidities. For children who were able to communicate verbally, caregivers also completed standardized rating scales to assess social competence and empathetic behavior as well as symptoms of hyperactivity/inattention and emotional problems.

**Results:**

As compared to children with idiopathic ASD, children with RASopathies were rated as demonstrating more resilience in the domain of empathy relative to their overall social competence. Similarities and differences emerged in the psychological factors that predicted social behavior in these two groups. Stronger communication skills and fewer hyperactive-impulsive behaviors were associated with increased empathy and social competence for both groups. Greater emotional challenges were associated with lower social competence for children with RASopathies and *stronger* empathy for children with idiopathic ASD. Among children with RASopathy and a co-occurring ASD diagnosis, socially empathetic behaviors were observed more often as compared to children with idiopathic ASD.

**Conclusions:**

Findings suggest that the development of social behavior among children with RASopathies involves a distinct pattern of strengths and weaknesses as compared to a behaviorally defined disorder (idiopathic ASD). Identification of areas of resilience as well as behavioral and social challenges will support more targeted intervention.

**Supplementary Information:**

The online version contains supplementary material available at 10.1186/s11689-021-09414-w.

## Background

The “RASopathies” are a set of clinically similar genetic syndromes that result from pathogenic variants within the RAS-MAPK pathway, a cellular signaling pathway whose functions include regulating growth factors and embryological development [1]. Children with RASopathies manifest physical, medical, and behavioral characteristics that vary widely in severity across individuals. Features spanning the RASopathy spectrum include short stature and growth issues, dermatological findings, cardiac disease, and increased risk for oncologic conditions. The two most common RASopathies are neurofibromatosis type 1 (NF1; estimated incidence of 1:3000 births) and Noonan syndrome (NS; 1:1000 to 1:2500), which are caused by alterations of genes encoding upstream components or regulators of RAS-MAPK signaling [2–4]. Less common RASopathies include cardiofaciocutaneous syndrome (CFCS; 1:810,000) and Costello syndrome (CS; 1:1.29 million), which are associated with dysregulation of molecules that function downstream in the signaling cascade [5]. Neuropsychological challenges occur more frequently in all RASopathies relative to the general population, including cognitive and learning disabilities; problems with attention, hyperactivity, and impulsivity; language impairments; and social and emotional problems [6–10]. There is considerable association between genotype and neurodevelopmental phenotype in RASopathies. CFCS and CS are frequently associated with mild to severe intellectual disability, whereas intellectual disability is infrequent (< 20%) in NF1 and NS [11–13].

Concerns within the social domain are widely observed across RASopathies, and clinical assessments indicate a higher than expected prevalence of autism spectrum disorder (ASD) symptomatology and diagnosis [6, 7, 14–17]. The nature of social behavior in RASopathies relative to ASD, a neurodevelopmental disorder defined by behavioral criteria, is nevertheless poorly understood [18]. Diagnosis of ASD requires the presence of impairments in social communication and interaction as well as restricted or repetitive activities, interests, or behaviors [19]. Population studies estimate that 1 in 54 children have ASD, with boys being four times as likely as girls to have this diagnosis [20]. Genomic sequencing suggests that more than 30% of ASD cases may be accounted for by chromosomal, de novo gene mutation and copy number variants [21]. Among “syndromic” forms of ASD, in which ASD presentation is associated with a known genetic syndrome, presentation of ASD symptomatology and other behavioral features can deviate to some extent from the typical presentation of idiopathic ASD (iASD), where no specific molecular pathology has been identified [22–24]. Defining the degree of convergence between “behaviorally defined” and “molecularly defined” social impairment is an important step toward determining whether and how specific biological pathways present risk for ASD symptomatology. Furthermore, an improved understanding of how social impairment arises in children with genetic syndromes will guide the development of more optimal screening and intervention approaches for these populations.

While an expanding number of studies are reporting an increased risk for social problems among individuals with RASopathies, the conceptualization of RASopathies as a form of “syndromic autism” [25] is likely an oversimplification for several reasons. First, a sizeable subgroup of children with RASopathies exhibit intact social behavioral characteristics, or even have a relative strength in social functioning despite pronounced intellectual or learning deficits [26–29]. Efforts to understand the *resilience* of social function in this subset of individuals may therefore be as instructive as focusing on individuals with more severe deficits. Second, individuals with milder or “subclinical” social impairment comprise another significant subset of children with RASopathies, and these children often do not meet diagnostic criteria for ASD on clinical evaluation [14]. Families of children with these milder symptoms may struggle to obtain resources to support better social functioning, since these symptoms may be perceived as secondary to deficits in other areas (e.g., cognitive impairment, ADHD). Access to potentially useful treatments (e.g., social skills training, early intensive behavioral intervention) often requires a clinical or educational diagnosis of ASD, which may not be applicable in these cases. Finally, while there is a subset of individuals with RASopathies who exhibit behavioral characteristics consistent with a clinical diagnosis of ASD, the extent to which these characteristics arise in the context of similar neurobehavioral patterns to iASD has only begun to be explored, with mixed results [6, 30].

Estimates of ASD prevalence among individuals with RASopathies have varied widely across studies. These estimates appear to depend heavily upon the specific assessment tools and methodology used as well as study sample characteristics [18]. Among clinically referred cohorts, ASD estimates in RASopathies are generally higher as compared to epidemiological samples [16]. Some evidence suggests that individuals with comorbid autism and RASopathy diagnoses (RAS+ASD) present with a distinct phenotype characterized by pronounced impairments in social communication (e.g., difficulty interpreting social cues), but fewer restricted and repetitive behaviors than typically observed in iASD [30]. Other research has failed to ascertain a unique RAS+ASD phenotype using quantitative caregiver report measures or ASD diagnostic instruments [6, 31].

Delineating the pattern of strengths and weaknesses across different components of social functioning is one approach that may be helpful in understanding how social development in RASopathies compares with iASD. Although most studies have focused on quantifying the increased risk of social deficits relative to unaffected peers, there is intriguing evidence that children with RASopathies may function relatively well in specific aspects of social functioning. In particular, studies have reported behaviors and personality characteristics that reflect a desire to be helpful, caring, or socially connected. Individuals with NS have been described to show a tendency toward friendliness, cooperativeness, and desire to please, reflecting a socially desirable attitude [26]. Additionally, children with NF1 have been found to be rated as more extraverted than their typically developing peers [32]. In a study investigating teacher and peer perceptions about children with NF1, students with NF1 were rated as displaying less leadership behavior and exhibiting greater social isolation and sensitivity than their age-matched peers. However, teachers notably rated children with NF1 as being *more* prosocial (e.g., polite, helpful to others) than other children in the classroom [27]. In this same study, children with NF1 rated their own prosocial behaviors as similar to their peers’ self-ratings. Longitudinal research has also identified a relative strength in social interest and social functioning in CS [28, 29]. Children with CS have been described to demonstrate such a consistent tendency toward friendliness and sociability that this personality feature is thought to be characteristic of the syndrome [33–35]. Thus, an observation of a friendly, helpful, or “prosocial” attitude has been described in multiple RASopathies. These findings suggest that relatively strong prosocial behaviors may be evident among children with RASopathies, even amid those who otherwise have considerable difficulty demonstrating social competence within interpersonal interactions.

Several previous studies of children with RASopathies or iASD have evaluated prosocial behaviors using the Strengths and Difficulties Questionnaire (SDQ [36, 37]), a brief measure that can be administered to various informants (e.g., parents, teachers, self-report) to screen social-emotional function. In general, research involving the SDQ has reported that children with NF1 are rated more positively with respect to their prosocial behaviors (behaviors intended to benefit others) as compared to the quality of their peer relationships (likability, friendships). In one study, 46% of parents who had a child with NF1 reported abnormalities on a scale assessing peer problems, whereas only 9% reported deficits in prosocial behaviors [38]. Another study found that although children with NF1 were five times as likely as healthy peers to have significant peer problems, the distribution of prosocial behavior ratings for children with NF1 across the normal, borderline, and abnormal ranges was very similar to the general population [39]. In terms of self-report among children with NF1, one study found that all affected children rated themselves as having normal prosocial behavior on the SDQ, while 14% reported abnormal levels of peer problems [40]. Overall, these findings suggest that prosocial deficits are reported to a lesser degree than peer relationship problems across raters.

The fact that multiple studies suggest relatively preserved prosocial behavior in the context of increased risk for social problems in RASopathies is curious given that RASopathies are associated with heightened risk of ASD, a disorder characterized by prosocial deficits [41, 42]. Children with even mild ASD symptoms are more likely to demonstrate prosocial deficits on the SDQ, and prosocial behavior scores can be used, along with emotional symptoms scores, to differentiate individuals with ASD from children with internalizing and externalizing disorders [43, 44]. Because the bulk of research measuring prosocial behavior in NF1 has relied on the SDQ, it is unclear to what extent these findings are dependent on the qualities of that specific rating scale. In a study of adults with NF1 using a different measure of social functioning (Social Performance Survey Schedule [45]), Pride et al. [46] found that despite normal self-ratings from individuals with NF1 regarding their prosocial tendencies, parents and friends observed weaker prosocial behaviors in these individuals.

Closely related to prosocial behavior is empathy. Blair [47] defines empathy as “an emotional reaction in the observer to the affective state of another” (p.669) and suggests that empathy can be divided into at least three types, including cognitive empathy (i.e., theory of mind), emotional/affective empathy (i.e., feeling an emotion someone else is feeling), and motor empathy (i.e., mirroring the expressions or gestures of another person). Though cognitive and affective empathy have been studied in RASopathies, motor empathy has not yet been examined. Cognitive empathy has consistently been found to be predictive of most types of prosocial behaviors in children, regardless of the cognitive task used [48]. Whereas prosocial behavior involves helping, sharing, cooperating, and comforting actions, cognitive empathy involves insight into other individuals’ thoughts, sometimes described as “theory of mind” [48]. As compared to the broad population of children with ASD, research indicates that children with NF1 + ASD have a stronger ability to comment on others’ emotions as well as insight into social situations and relationships [49]. However, some studies have found deficits in measures of cognitive empathy and perspective-taking. Payne et al. [50] found that children with NF1 performed similarly to unaffected children when determining the sequence of physical cause-and-effect stories, but they showed comparative difficulty determining the correct sequence for stories that required theory of mind skills. In another study, adults with NF1 showed less empathy and had more difficulty making social inferences than controls [51]. In a study of adults with NS, Wingbermuhle et al. [9] reported that performance on a theory of mind task was similar in the individuals with NS as compared to unaffected adults. Results regarding emotional recognition skills for individuals with NS have been mixed. Roelofs et al. [52] found no overall difference between women with NS and controls in their ability to identify emotions in facial expressions (other than that women with NS had more difficulty recognizing anger), whereas other studies suggest slightly lower emotional identification skills in adults with NS [9, 26]. Thus, findings regarding prosocial behavior and empathy in RASopathies are mixed and warrant further investigation.

In addition to measuring these more specific aspects of social behavior, consideration of whether similar neuropsychological factors and comorbidities moderate the expression of social behaviors in RASopathies as compared to iASD may be useful. This approach may clarify how dysregulation of RAS-MAPK signaling can contribute to development of ASD among some affected individuals, and to social challenges more broadly in a larger subset. Investigations aiming to identify neuropsychological predictors of social problems in RASopathies have increased in recent years, although most studies have focused on individuals with NF1. There is overwhelming evidence that attention problems and executive functioning deficits increase the risk of social problems and ASD symptoms among children with RASopathies [6, 15, 38, 53–58]. Aspects of communication, including structural and pragmatic language deficits, have also been found to predict social deficits [15, 59]. A systematic review by Chisholm et al. [53] found that perceptual and higher-level impairments in social cognition contribute to social outcomes in NF1, and there is initial evidence that emotional difficulties (e.g., social anxiety) may also impact social competence in NF1 [60]. Examination of the extent to which some of these key neuropsychological features vary across the RASopathies and iASD, and whether they are differentially associated with social behavior, may provide insights that could lead to more effective treatment strategies in these populations.

To test the hypotheses that social behavior, and the neuropsychological correlates of social behavior, differ between children with RASopathies and those with iASD, the current study utilized parent/caregiver rating scales to focus on two components of social behavior: social competence and prosocial (empathetic) behavior. Whereas social competence refers to the degree of effectiveness in social interactions, prosocial behavior refers to social behaviors that are meant to benefit others [61, 62]. Due to limited availability of measures of prosocial behavior, we broadened this outcome to include empathy, a construct closely linked to prosocial behavior [47, 48]. The measure selected to assess empathy, the Social Emotional Assets and Resilience Scales (SEARS [63];), included questions capturing both cognitive and affective empathy. The first goal of the current study was to assess social competence and empathy among school-aged children with RASopathies relative to iASD. A secondary goal was to determine whether key neuropsychological variables (i.e., hyperactivity/inattention, emotional symptoms, and communication problems) were predictive of social competence and empathetic behavior for individuals with RASopathies and iASD and to compare these patterns between groups. The final goal was to examine these patterns specifically for individuals with RASopathy and a co-occurring ASD diagnosis (RAS+ASD) relative to those with iASD.

## Methods

### Participants

**Fig. 1:**
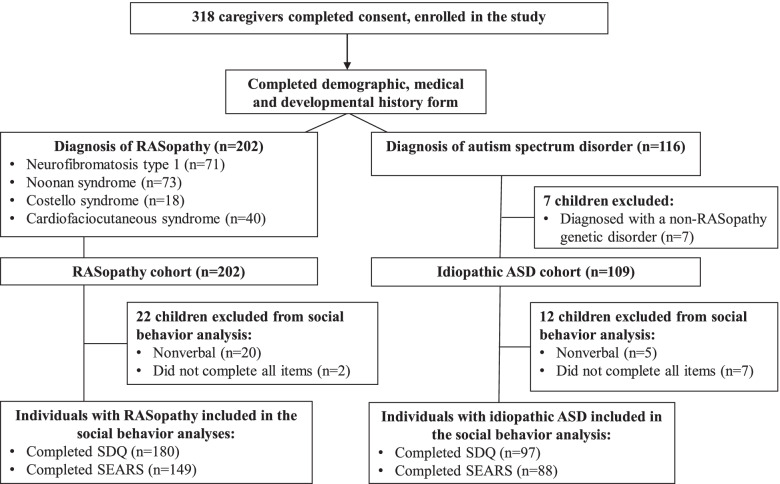
Flow diagram for study participants

Study participants were recruited via email/listservs (39%), social media (34%), conferences (CFC International, Costello Syndrome Family Network annual meeting; 16%), clinic appointments (9%), flyers (2%), or someone else in the community (1%). Parents/caregivers of individuals with NF1, NS, CFCS, CS, and ASD were invited to participate. The majority of respondents were mothers (93%), followed by fathers (7%), grandmothers (1%), and aunts (< 1%). Families originated from the following countries: USA, Canada, Australia, and New Zealand. Figure 1 contains a flow chart of participants enrolled in the study. A total of 318 caregivers of children with a RASopathy diagnosis and/or an ASD diagnosis completed at least one portion of the survey. Seven participants in the ASD group were excluded from analysis due to having identified non-RASopathy genetic variants. The remaining sample included 311 children with RASopathies (*n* = 202) or iASD (*n* = 109). Children ranged in age from 3.0 to 17.7 years (mean age 9.8 years; SD 4.1 years). The diagnosis of a RASopathy was confirmed by molecular testing in most cases (Table 1). The cohort included 179 boys and 130 girls. One participant in the NF1 group and one participant in the iASD group identified as having non-binary gender.

**Table 1:** RAS-MAPK genes affected among participants with RASopathies

	Molecular testing % (*N*)	Confirmed variants among those tested
CFCS	100% [41]	BRAF (60%) MAP2K1 (MEK1) (28%) MAP2K2 (MEK2) (5%) YWHAZ (5%) Unknown (3%)
CS	100% [18]	HRAS (100%)
NF1	70% [50]	NF1 (98%) Unknown (2%)
NS	93% [64]	PTPN11 (57%) RAF1 (9%) SOS1 (9%) KRAS (6%) RIT1 (4%) SOS2 (3%) BRAF (3%) PPP1CB (2%) A2ML1 (1%) Unknown (6%)

After initial demographic, medical, and developmental background data were collected, surveys regarding social behaviors were presented to caregivers of children who had the ability to communicate verbally. Based on responses to developmental questions in a brief adaptive measure [65], social behavior surveys were not administered to caregivers of 25 children in the study who lacked functional verbal communication or language comprehension, as the measures were not deemed appropriate for children who are nonverbal. An additional 7 participants stopped the surveys before completing all of the items. The remaining 277 participants with RASopathies (*n* = 180) or iASD (*n* = 97) completed the SDQ. Due to a more restricted normative age (i.e., children ≥5 years), a slightly smaller cohort (*n* = 237) completed the Social Emotional Assets and Resilience Scales (SEARS [63]). Given these exclusions, children with RASopathies and/or ASD included in the social behavior analyses had somewhat better communication skills and were slightly older than the cohort as a whole.

### Study design

Families interested in participating were provided a secured, private link to the study questionnaires, which were administered to caregivers electronically through REDCap (Research Electronic Data Capture [66]). The research protocol was approved by the University of Minnesota Institutional Review Board and the University of Wisconsin-Madison Institutional Review Board. Survey responses were collected between July 2019 and March 2020. Only one survey (from a parent of a child with CFCS) was completed after the onset of the COVID-19 pandemic.

### Measures

#### Background history form

Parents provided information about the child’s age, gender, medical history, and family demographics. This form also collected information about whether any of the following psychiatric diagnoses had been assigned in a medical setting by a qualified health care provider (e.g., a physician or psychologist): ADHD, ASD, anxiety, depression, or intellectual disability.

#### Communication ability

Communication abilities were estimated using the Great Outcomes for Kids Impacted by Severe Developmental Disabilities (GO4KIDDS) Brief Adaptive scale, a short measure of adaptive function validated for individuals with intellectual disabilities [65]. This measure has high internal consistency as well as strong convergent validity (*r* > 0.80) with the Vineland Adaptive Behavior Scales, Second Edition and the Scales of Independent Behavior-Revised form [65, 67]. Items were rated on a 5-point scale, with higher scores indicating stronger communication abilities and lower scores indicating minimal ability to use or understand language. A composite score based on the receptive and expressive communication items estimated each child’s functional communication ability.

#### Social functioning

*The Social Emotional Assets and Resilience Scales- Parent* (SEARS-P [63]) measures the social-emotional competencies and assets of children and adolescents 5–18 years old. Respondents rated children with regard to various social skills and characteristics on a 4-point Likert scale (“never,” “sometimes,” “often,” “always”), with higher scores indicating greater competency. Caregivers were administered two scales that measured Social Competence (i.e., “ability to create and maintain peer friendships, ability use verbal communication effectively, comfort in group situations with peers”; 10 items) and Empathy (i.e., “ability to relate to and understand the experiences and emotions of others”; 7 items). SEARS scores are reported in *T*-scores with a mean of 50 and a standard deviation of 10, with higher scores indicating fewer problems. The Empathy and Social Competence scales have demonstrated both strong internal consistency reliability and inter-rater reliability [63]. The SEARS-P has shown convergent validity with other informant rated strengths-based measures of social-emotional competence, including the Social Skills Rating System (SSRS [68]) and the Home and Community Social Behavior Scales (HCSBS [63, 64]).

The *Strengths and Difficulties Questionnaire* (SDQ; 36, 37) is a widely used behavioral screening questionnaire assessing concerns across five domains: Emotional Symptoms, Conduct Problems, Hyperactivity/Inattention, Peer Relationship Problems, and Prosocial Behavior. Items are rated on a three-point scale (“not true,” “somewhat true,” “certainly true”) to indicate severity of behavioral challenges. The Prosocial Behavior scale reflects the ability to demonstrate behaviors intended to help or show caring for others (e.g., “Kind to younger children”). This scale was reverse-scored in our study to be consistent with the other 4 scales, so that higher scores would indicate more problems and concerns consistent with a lack of prosocial behavior. SDQ scores are reported in *z*-scores with a mean of 0 and a standard deviation of 1. The five-factor structure has been confirmed, and the measure correlates highly with diagnostic categories and other measures of internalizing and externalizing behaviors in children [36, 69–71]. The SDQ has strong predictive validity and acceptable levels of test-retest-reliability (0.62 for 4–6 months [69, 71]). Internal consistency reliability across different SDQ scores and informants is also satisfactory (mean Cronbach 0.73 [71]). Table S1 (Additional file) contains more detailed descriptions of the SEARS and SDQ scales.

### Statistical methods

Descriptive statistics delineate the medical complications and comorbidities present in the study sample. A few data points were missing for items assessing visual impairment (*n* = 2) and hearing impairment (*n* = 2). For the analysis of social behavior, within- and between-group differences in scores on the Social Competence and Empathy scales of the SEARS were evaluated using within-subjects ANOVA. Standardized scores were used for all group comparisons except the GO4KIDDS communication composite, for which no age-based normative scores are available. For all measures, clinically significant deficits were defined as ≥2 SD below the normative sample mean. Multiple linear regression was performed to model the relationship between three pre-specified predictor variables (SDQ Emotional Symptoms scale; SDQ Hyperactivity/Inattention scale; and GO4KIDDS Communication composite score) and the two social behavior outcome variables (SEARS Social Competence and Empathy scales). The relationship between the set of predictor variables and the two outcome variables was examined separately for the RASopathy group (comprised of children with NF1, NS, CFCS, and CS) and the iASD group, resulting in four separate regression models. Statistical analyses were performed using IBM SPSS Statistics 26 package.

## Results

### Child characteristics and comorbidities

Data regarding medical history were collected using the background questionnaire completed by caregivers (Table 2). In terms of medical complications, children with CFCS were by far the most likely to experience seizures, whereas tumors were most frequent in children with NF1. Vision impairment was common across RASopathies, while hearing impairment was most common in NS and CFCS. Children with NS or CFCS were the most likely to report other medical complications (e.g., heart condition, motor impairment, sleep disorders). Preterm birth was also more common in these two syndromes. Children with iASD were more likely to be male, consistent with the gender distribution of ASD in the general population [20].

**Table 2:** Demographics of study participants

	NF1 (*n* = 71)	NS (*n* = 73)	CFCS (*n* = 40)	CS (*n* = 18)	RASopathy (*n* = 202)	iASD (*n* = 109)
Participant demographics						
Age of child (M (SD))	9.8 (4.37)	9.2 (4.21)	8.8 (4.10)	11.0 (4.21)	9.5 (4.26)	10.4 (3.67)
Gender (*N* (%) male)	36 (51)	37 (51)	17 (43)	9 (50)	99 (49)	80 (73)
Race (*N* (%))						
American Indian or Alaska Native	0 (0)	1 (1)	1 (3)	0 (0)	2 (1)	5 (5)
Asian	2 (3)	4 (6)	3 (8)	2 (11)	11 (5)	5 (5)
Black or African American	4 (6)	3 (4)	1 (3)	0 (0)	8 (4)	5 (5)
Native Hawaiian or Other Pacific Islander	1 (1)	1 (1)	0 (0)	2 (11)	4 (2)	2 (2)
White	65 (92)	69 (95)	37 (93)	17 (94)	188 (93)	96 (88)
Other	1 (1)	1 (1)	2 (5)	2 (11)	6 (3)	6 (5)
Ethnicity (% Hispanic/Latinx)	13 (18)	4 (6)	3 (8)	1 (6)	21 (10)	15 (14)
Medical complications (*N* (%))						
Preterm birth	6 (9)	12 (16)	13 (33)	8 (44)	39 (19)	9 (8)
Seizures	10 (14)	7 (10)	20 (50)	2 (11)	39 (19)	1 (1)
Tumor	31 (44)	4 (6)	1 (3)	0 (0)	36 (18)	0 (0)
Visual impairment	20 (29)	37 (51)	29 (73)	12 (67)	98 (49)	20 (19)
Hearing impairment	4 (6)	16 (22)	10 (25)	0 (0)	30 (15)	2 (2)

Psychiatric comorbidities were common among children with RASopathies as well as those with iASD (Table 3). Based on prior studies estimating prevalence of psychopathology among children with RASopathies using diagnostic methods or questionnaires, the diagnosis of ADHD was less common than expected in our cohort for children with NS, CFCS, and CS. Diagnosis of anxiety was slightly higher than expected for NF1, NS, and CFCS. The comorbidities reported among children with iASD in this sample were within the expected range for all comorbidities, with approximately half having been diagnosed with ADHD and more than a third having anxiety. Individuals with RASopathies were more likely to have been diagnosed with intellectual disability than individuals with iASD. Within RASopathies, an intellectual disability diagnosis was much more frequent in CFCS and CS than in NS and NF1 (Table 2), consistent with previous literature [11]. It is important to note that these numbers may underestimate the prevalence of intellectual disability or ADHD, given the young age of some of the participants and the difficulty that some affected children have with participating in standardized cognitive testing. In terms of language abilities, caregiver ratings of receptive/expressive communication skills (GO4KIDDS composite) were similar or slightly weaker in the iASD group as compared to the RASopathy group (mean difference = 0.3, 95% CI − 0.1 to 0.6).

**Table 3:** Psychiatric and neurodevelopmental diagnosis prevalence estimates in the literature as compared to this study cohort (%)

	NF1 (*n* = 71)	NS (*n* = 73)	CFCS (*n* = 40)	CS (*n* = 18)	iASD (*n* = 109)
**ASD**					
Estimates	11–26 [16, 30, 38, 53]	0–30 [7, 14]	9–54 [6, 7]	11–26 [6, 7]	100
Our study	16	10	23	6	100
**ADHD**					
Estimates	39 [72]	34–48 [10, 14]	47–89 [14, 73]	38 [7]	28–76 [74, 75]
Our study	41	25	15	6	47
**Anxiety**					
Estimates	18–21 [72, 76]	7–8 [72, 77]	6 [73]	20–50 [12]^*^	32–79 [78, 79]
Our study	18	14	28	22	37
**Depression**					
Estimates	–	–	12 [73]	–	1–56 [74, 75]
Our study	11	6	0	0	13
**Intellectual disability**					
Estimates	4–8 [80, 81]	6–23 [10, 82]	90–100 [5, 83, 84]	78–100 [5, 12, 29]	33 [20]
Our study	10	15	63	67	11

– No research available; *for males (primarily separation anxiety)

### Social behavior analysis

Demographics of the subset of children with verbal communication whose caregiver completed at least one social behavior survey and were included in the following analysis are available in supplemental Table S2.

#### Comparison across scales assessing social competence and empathy

For 149 children with RASopathies and 87 children with iASD who were in the 5–17-year-old age range, data were available for both the Social Competence and Empathy scales of the SEARS. Examination of between-group differences along the two SEARS scales indicated higher group means for both Social Competence and Empathy for children with RASopathies as compared to children with iASD, with standard deviations indicating somewhat larger variance in the RASopathy group (Table 4).

**Table 4:** Social and behavioral ratings of children with RASopathies or idiopathic ASD

	RASopathy				Idiopathic ASD					
Measure	*n*	Mean	(SD)	% Clinical impairment (≥ 2 SD below mean)	*n*	Mean	(SD)	% Clinical impairment (≥ 2 SD below mean)	Mean difference (95% CI)	
**Social Emotional Assets & Resilience Scales (SEARS)**										
**Social competence**	149	39.57	(11.10)	19	87	30.49	(7.64)	48	9.08	6.43 to 11.73
**Empathy**	149	45.30	(11.16)	8	88	32.99	(9.39)	40	12.31	9.52 to 15.10
	***n***	**Mean**	**(SD)**	**% Clinical impairment (≥ 2 SD above mean)**	***n***	**Mean**	**(SD)**	**% Clinical impairment (≥ 2 SD above mean)**	**Mean difference (95% CI)**	
**Strengths & Difficulties Questionnaire (SDQ)**										
**Emotional symptoms**	180	1.23	(1.45)	29	97	1.32	(1.29)	29	− 0.09	− .44 to .26
**Conduct problems**	180	0.48	(1.15)	11	97	0.71	(1.10)	17	− 0.23	− .51 to .06
**Hyperactivity/inattention**	180	1.33	(1.15)	32	97	1.59	(1.03)	46	− 0.26	− .54 to .01
**Peer relationship problems**	180	1.33	(1.42)	38	97	2.21	(1.11)	58	− 0.87	− 1.2 to − .55
**Lack of prosocial behavior**	180	0.83	(1.25)	22	97	1.79	(1.24)	44	− 0.96	− 1.27 to − .65

SEARS scores are reported in *T*-scores with a mean of 50 and a standard deviation of 10. Higher SEARS scores indicate fewer problems; SDQ scores are reported in *z*-scores with a mean of 0 and a standard deviation of 1. Higher SDQ scores indicate more problems

Within-subjects ANOVA was used to compare caregiver ratings across the two SEARS scales (Social Competence vs. Empathy) in the RASopathy and iASD groups. Results showed evidence of interaction between the scales and the diagnostic group (F_1, 234_ = 6.34; *p* = 0.01), with a larger discrepancy between the Social Competence and Empathy scales in the RASopathy group as compared to the iASD group. For both groups, average ratings of Empathy were higher than Social Competence. However, the difference favoring Empathy was more than twice as large for children with RASopathies (mean difference 5.7 points) than for children with iASD (mean difference 2.5 points). Further, 11% of children in the RASopathy group had above average (> 1 SD above normative average) Empathy scores as compared with 3% in the iASD group.

**Fig. 2:**
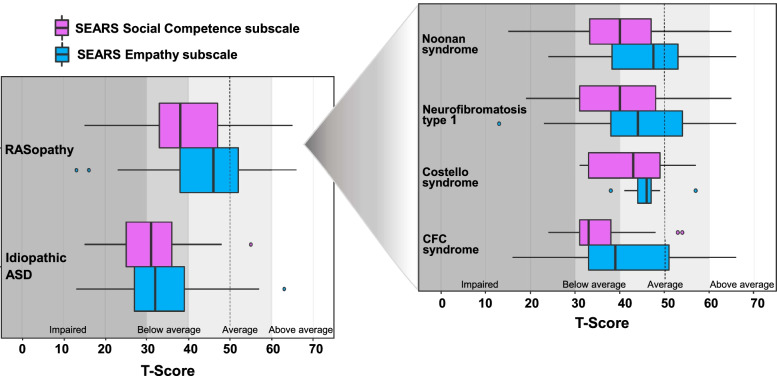
Social Competence and Empathy scores for children with RASopathies and children with idiopathic ASD

In terms of comparison to population norms, the median score for Social Competence was in the below average range for children with RASopathies, whereas the median score for the Empathy scale was within the average range. When separated out by specific RASopathy diagnoses, the observed pattern of higher Empathy scores relative to Social Competence scores was seen consistently across all four RASopathies (Fig. 2). For the iASD group, median scores for Social Competence and Empathy were both below average.

Standardized scores for the SDQ scales were compared between the RASopathy and iASD groups, with a slightly larger (and younger) sample than for the SEARS (Table 4). Similar to results for the SEARS, the RASopathy group had notably stronger scores on both of the SDQ social behavior scales than the iASD group. The greatest difference between the RASopathy and iASD groups was on the Prosocial Behavior scale (mean *z*-score difference = − 0.96), in which the RASopathy group scored nearly one standard deviation higher that the iASD group relative to normative samples. Scores on the Peer Relationship Problems scale were also notably higher in the RASopathy group (mean *z*-score difference = − 0.88). In contrast, the severity of emotional challenges in the RASopathy group was similar to the iASD group. Scores on the Emotional Symptoms scale did not differ meaningfully between these groups (mean *z*-score difference = − 0.09), although it is important to note that this scale also showed the greatest variance for both groups. Conduct Problems and Hyperactivity/Inattention were mildly less problematic for the RASopathy group than for the iASD group.

Examination of the social behavior scales when separated out into the four individual RASopathy groups (Tables S3-S4) revealed that in all RASopathies but CS, children were more likely to have clinical deficits in the Social Competence domain than in the Empathy domain. This pattern was also true for the iASD group. However, the proportional difference among these two scores was somewhat larger for RASopathies. Children with NF1 and NS showed the largest mean differences in *T*-scores between the Social Competence and Empathy scales (mean differences: 5.9 and 6.1 points for each group, respectively) relative to children with CFCS and CS (mean differences: 5.3 and 3.9). For all RASopathies, the difference between mean Social Competence and Empathy ratings were larger than for the iASD group. No children with CS were rated as having clinical impairment on either the Social Competence or Empathy scales, though this sample was the smallest. Children with iASD were much more likely than any RASopathy group to have deficits on the SEARS scales.

#### Predictors of social behavior

Multiple linear regression models examined predictors of the two components of social behavior as measured by the SEARS (Table 5). For the RASopathy group, the three predictors (SDQ Hyperactivity/Inattention, SDQ Emotional Symptoms, GO4KIDDS Communication Composite) accounted for similar proportions of variance in each of the two social behavior outcomes scales. Specifically, the predictors accounted for 24% (*R*^2^ = .237) of variance in Social Competence scores and 23% (*R*^2^ = .231) of variance in Empathy scores. In the iASD group, the three predictors accounted for 11% (*R*^2^ = .114) of variance in Social Competence scores and 28% (*R*^2^ = .283) of variance in Empathy scores.

**Table 5:** Results of multivariable regression models for prediction of SEARS Social Competence and Empathy scales

	RASopathy (***n*** = 149)		Idiopathic ASD (***n*** = 88)	
Model	Regression coefficient	(95% CI)	Regression coefficient	(95% CI)
**Model 1: Factors predicting social competence**				
**Intercept**	34.86	(22.78 to 46.93)	19.94	(10.55 to 29.33)
**Emotional symptoms**	− 2.55	(− 3.78 to −1.33)	− 0.17	(− 1.5 to 1.17)
**Hyperactivity/inattention**	− 1.87	(− 3.35 to − .38)	− 0.82	(− 2.49 to .84)
**Communication composite**	1.21	(− .04 to 2.45)	1.42	(.41 to 2.43)
**Model 2: Factors predicting empathy**				
**Intercept**	19.75	(7.57 to 31.93)	16.64	(6.31 to 26.97)
**Emotional symptoms**	0.80	(− .43 to 2.04)	1.83	(.35 to 3.31)
**Hyperactivity/inattention**	− 2.43	(− 3.93 to − .93)	− 3.01	(− 4.85 to −1.18)
**Communication composite**	3.12	(1.86 to 4.38)	2.18	(1.07 to 3.30)

In terms of predictors of Social Competence, children who had stronger communication abilities tended to have higher Social Competence scores across the diagnostic groups (Table 5). Greater presence of Hyperactivity/Inattention symptoms was also predictive of lower Social Competence for the RASopathy and iASD groups, though this effect was larger in the RASopathy group. The impact of Emotional Symptoms scores contrasted markedly between groups, with little overlap in confidence intervals. Children with RASopathies with greater Emotional Symptoms had worse Social Competence scores, whereas this relationship was not evident for the iASD group.

Across the RASopathies and iASD groups, similar predictors were associated with empathetic behaviors. Communication ability accounted for the greatest amount of variance in the Empathy scale for both the RASopathy and iASD groups, such that children with better verbal abilities showed higher Empathy scores. This effect was slightly stronger for the RASopathy group than for the iASD group. Symptoms of Hyperactivity/Inattention were also associated with poorer Empathy scores in both groups. Curiously, greater anxiety/mood disturbance was associated with *stronger* Empathy for the iASD group.

#### Impact of ASD diagnosis on social behavior in RASopathies

In our cohort, 22 (14%) of children with RASopathy had received a diagnosis of ASD (RAS+ASD) from a healthcare professional. To determine whether trends in Social Competence and Empathy differed among participants with RAS+ASD versus those with iASD, scores on the SDQ and SEARS scales were compared between these groups (Table S5). While verbal participants with RAS+ASD had a similar risk of severe deficits in social competence relative to those with iASD (45% vs 48%), they were less likely to have severe deficits in Empathy (25% vs 40%). Of the SDQ scales, the greatest difference between groups was seen in Emotional Symptoms; verbal children with RAS+ASD were more likely to have clinical impairment on this scale than those with iASD (41% vs 29%).

## Discussion

In this investigation, we sought to examine the potential overlap between comorbidities, social behavioral patterns, and predictors of social behavior in children with RASopathies as compared to children with a behaviorally defined diagnosis of ASD (i.e., idiopathic ASD or ‘iASD’). In terms of the rate of comorbidities between RASopathies and iASD, our sample as a whole was generally consistent with estimates from the literature, although somewhat fewer children with CS than expected had an ASD diagnosis [6, 7]. As far as comorbidity with ADHD, fewer children with NS, CFCS, or CS had been diagnosed with ADHD than expected [10, 14, 75]. A potential explanation for this finding is that clinicians may view attentional concerns as secondary to the more global developmental delays or intellectual disability that can sometimes occur in these groups, and therefore conclude that an additional ADHD diagnosis is not warranted. Other possibilities are that the symptom rating scales often used in research studies may overestimate the true prevalence of clinical ADHD, or that the young age of many children in the study may have precluded a diagnosis of ADHD, as some clinicians do not consider this diagnosis to be appropriate prior to age 6 (23% of our study participants were < 6 years old). When looking at emotional concerns, more children with NS and CFCS had been diagnosed with anxiety than anticipated based on the rate of symptoms reported in past studies [72, 77]. Surprisingly few studies have reported the rate of depression among children with RASopathies, rendering a comparison with expected prevalence rates more challenging.

Another focus of the current study was to examine patterns of strengths and weaknesses within the social domain. Our results provide evidence that despite overall deficits in social behavior, many or most children with RASopathies exhibit a relative strength in empathetic, helpful behaviors that demonstrate a desire to please others. The difference between the ability to manage interpersonal interactions effectively (i.e., social competence) and the ability to demonstrate empathy was markedly greater for children with RASopathies than for children with iASD. This finding of relatively strong empathy was also observed when comparing children with RASopathy who had a co-occurring ASD (RAS+ASD) diagnosis to children with iASD.

Evidence of a distinction between the social behavior patterns observed in RAS+ASD versus iASD is evident in the literature on NF1; studies have reported that NF1 + ASD may be characterized by fewer stereotyped behaviors, better eye contact, and stronger language skills than iASD [30, 49, 85–87]. In the present study, stronger language skills were linked to improved empathy and social competence in both diagnostic groups, but the link between language and empathy was particularly strong in the RASopathy group. Although one might hypothesize that stronger language skills in participants with RAS+ASD as compared to those with iASD in the current study contributed to the finding of stronger empathy skills, this explanation is not likely given that participants with RAS+ASD were rated as having poorer communication skills on average as compared to participants with iASD. Another contributing factor to differences in the RAS+ASD and iASD social behavior profiles could be the attenuated male-to-female bias seen in the RAS+ASD group. Individuals with iASD are 3–4 times as likely to be male than female, whereas individuals with RASopathies who have clinical presentation of ASD symptoms are 2–3 times as likely to be male than female [6, 20, 31, 86, 88]. In the current study, the male-to-female ratio for participants with RAS+ASD was 2.13, whereas the male-to-female ratio for participants with iASD was 2.85. Taken together, these results suggest that differences in overall communication skills or gender distribution could contribute to the distinct social behavior profiles identified in this study (i.e., stronger empathy among the RAS+ASD group as compared to the iASD group), but may not fully explain the observed patterns.

Our finding of relatively strong empathy in children with RASopathies stands in apparent contrast to a recent study of personality characteristics in this population [89]. In that study, verbal individuals with RASopathies were rated by parents as having lower scores on traits involving “agreeableness” and “conscientiousness” as compared to sibling controls [89], suggesting that prosocial tendencies were less well-developed in those individuals. Interestingly, an item analysis reported in the study revealed that children with RASopathies tended to have lower ratings on questions assessing what they were able to easily do, or quickly understand (e.g., “understands when help is needed”) as compared to items assessing their openness, kindness, or willingness to engage. These findings suggest that cognitive or communication challenges could cause a child to be rated as less empathetic or agreeable in survey results. For example, cognitive impairments may cause a child to have difficulty quickly processing or remembering information during interactions, and communication deficits can make it more difficult for a child to understand and produce language needed to demonstrate empathy. Indeed, the ability to demonstrate helpfulness and show recognition of the needs of others may be more difficult for a child who is less mobile, less able to function independently, and less able to communicate effectively. Consistent with this interpretation, our findings indicated that ratings of empathy and social competence were consistently associated with communication skills in both RASopathy and iASD groups. This conclusion aligns well with previous evidence supporting the notion that expressive language and pragmatic communication problems are important predictors of social deficits in RASopathies [15, 59]. A relationship between communication and social functioning has also been previously established for individuals with ASD, as early childhood language problems were found to be predictive of poorer social functioning among adults with ASD [90].

Aside from communication skills, our study investigated the relationship between two other psychological characteristics as predictors of social behavior in RASopathies and iASD: hyperactivity/inattention and emotional problems. Results indicated that greater symptoms of hyperactivity and inattention were associated with poorer social competence and empathy for both groups, although there was a slightly stronger relationship between hyperactivity/inattention symptoms and social competence for the RASopathy group. Numerous other studies have shown that ADHD diagnosis and symptomatology are associated with social problems among children with RASopathies [38, 91], and a recent study by Geoffray et al. [31] suggests an even higher prevalence of comorbid ADHD among children with NF1, NS, and CFCS as compared to children with iASD. These results lend support to the suggestion made by Chisholm et al. [53] that the increased risk of ASD traits in RASopathies may arise from a multifactorial basis inherent to the RASopathy phenotype as opposed to ASD-specific factors. The relatively high ADHD burden and the strengths in empathy we observed for individuals with RASopathies may represent distinct factors that lead to the social behavioral profiles of RASopathies versus iASD.

Although ADHD symptoms may not be as tightly linked to social deficits in iASD as in RASopathies, these symptoms have been linked to poorer social functioning for children with iASD as well. Previous research has found that social communication and social awareness, but not social motivation, tend to be associated with ADHD symptoms for children with iASD [92, 93]. Thus, ADHD symptoms appear to interfere with the display of social behavior quite broadly for children with RASopathy and iASD, although they do not seem to have an equivalent impact on all types of social behaviors. Inattentive symptoms, for example, may cause particular difficulty with noticing and attending to the needs of others and consistently participating during social interactions. Hyperactive/impulsive symptoms in turn may manifest as a higher frequency of annoying, aggressive, or disruptive behaviors that can affect a child’s likeability and acceptability among peers [94].

A starker contrast between the RASopathy and iASD groups emerged for the effect of emotional symptoms on social behavior. For the RASopathy group, children with greater emotional challenges (anxiety, mood disturbance) displayed poorer social competence. Previous research examining the role of anxiety in social problems for children with a RASopathy diagnosis has yielded mixed findings. While there is some evidence that social anxiety is associated with weaker social competence for children with NF1, other research has failed to find an association between increased overall anxiety and social competence for children with NF1 or NS [15, 60]. The relationship between emotional symptoms (primarily anxiety symptoms) and social competence in our study was complex. Higher levels of emotional challenges were associated with weaker social competence for RASopathy and, surprisingly, stronger empathetic behavior for iASD. In this regard, we speculate that a higher general level of emotional expression in a child with RASopathy may interfere with forming social relationships; these internalizing symptoms may result in social withdrawal [10, 60, 78]. In contrast, higher emotional burden in ASD seems to *facilitate* expression of thoughtful or caring gestures toward others (i.e., expression of empathy) in this sample. Interestingly, intervention supporting the verbal expression of empathy in individuals with ASD has been found to improve general empathy and increase individual’s confidence in their communication skills [95].

### Implications for intervention

For children with RASopathies or iASD in this study, unique patterns of social strength and weakness emerged that were associated with somewhat divergent underlying neuropsychological variables. Interventions intended to support better social functioning for children with these disorders may therefore need to be designed to target the specific neuropsychological factors that contribute to the social presentation demonstrated by any given child. Given the relative strength in empathetic behavior demonstrated by children with RASopathies in this study, it will be important for caregivers, educators, and clinicians to reinforce this area of strength by continuing to recognize, praise, support, and encourage the development of empathy skills for these children. Empathetic behavior is a significant resiliency factor that warrants continued study in children with RASopathies. Although empathy is a complex, multifaceted construct, the expression of empathy is undoubtedly important to the social success of children, fostering the development of positive peer relationships. Further research examining the role of empathy in development for children with RASopathies will be important for this reason.

In terms of addressing areas of challenge, children with iASD and those with RASopathies who have ASD and/or more limited cognitive and language abilities will likely benefit most from intensive behavior interventions (e.g., applied behavior analysis therapy) with particular emphasis in improving functional communication and adaptive skills. For children with CFCS and CS, communication abilities were more closely related to ASD status than for children with NF1 and NS. As such, communication-focused interventions will be especially important for children with CFCS and CS to support social functioning. Children with CFCS had the weakest communication skills and social competence of the RASopathy groups. Neurological comorbidities, including a heightened risk for seizures (in this study 49% for CFC, 10–14% for other RASopathies [96];), may in part explain this finding. These seizures, particularly infantile spasms and treatment-resistant epilepsy, can have detrimental impacts on neuropsychological function. Children with RAS+ASD were also more likely than children with iASD to have elevated levels of emotional symptoms, and they had weaker communication skills on average than children with RASopathy alone. Thus, addressing emotional symptoms and teaching emotional regulation skills may lead to improved social competence for children with RAS+ASD. Children with NF1 and NS were more likely than children with CS and CFCS to have emotional difficulties, suggesting that they may benefit the most from this intervention approach.

For a substantial subgroup of children with RASopathies, treatment of ADHD symptoms should be a primary target of intervention for social problems. In particular, intervention focusing on attentional and inhibitory control has been shown to improve children’s social competence [97, 98]. Further, children with ADHD in the general population are known to be at increased risk of problems with emotional regulation. Because emotional symptoms and hyperactivity/inattention problems were both associated with reduced social competence for children with RASopathy, interventions that support both emotional and behavioral regulation will be important for children with co-occurring RASopathy and ADHD diagnoses. Although executive functioning was not specifically measured in this study, interventions with a focus on teaching self-regulation skills may reduce the degree to which emotional problems and symptoms of hyperactivity/inattention impact social competence for children with RASopathies. This type of intervention is also likely to benefit children with iASD and RAS+ASD, as these children are at increased risk of ADHD symptoms and emotional difficulties. Finally, children with RASopathy and ADHD may benefit from medication management for ADHD symptoms, as this intervention approach has shown positive impacts on social functioning for children with NF1 diagnosed with ADHD [91].

Another potentially important target of intervention is social cognition. Prior literature presents substantial evidence that individuals with iASD have deficits with regard to their empathic understanding and that instruction in the verbal expression of empathy can be an effective intervention for adults with iASD [42, 95]. Although children with RAS+ASD and iASD in this study were at similar risk of social competence problems, a much larger proportion of the iASD group demonstrated a lack of empathetic/prosocial behavior. Interventions have been successful in strengthening both lower- and higher-level social cognitive abilities in individuals with iASD [99–101]. Preliminary evidence also suggests that social cognitive training can benefit adults with NS [102]. However, the few studies that have examined social cognition in individuals with RASopathies have yielded mixed findings, and additional research is needed to understand whether these types of interventions would be effective for children or adolescents with RASopathies [50, 56, 103].

Since the various influences on social behavior appear to be multifactorial, and RASopathies and iASD are highly heterogeneous disorders that result in a wide array of presentations, behavioral treatments that support development *across* the different areas of risk may hold greater promise than those focusing on a single domain in isolation. The Affective-Behavioral-Cognitive-Dynamic (ABCD) model of development proposed by Greenberg and Kusché [104] emphasizes the importance of the developmental integration of four factors: cognition, affect and emotional language, communication, and outward behavior. Furthermore, Riggs et al. [97] found evidence that a focus on integrating executive functions, verbal processing, and emotional awareness can lead to stronger social-emotional functioning and prosocial behavior. The current findings suggest that a focus on the combination of communication skills, emotional language, and executive functioning (behavioral and emotional regulation) may be an effective intervention approach for children with RASopathies and iASD who struggle with social competence. This finding also fits with RASopathy-specific models, such as the SOCIAL model originally developed by Beauchamp and Anderson [105] and adapted by Chisholm et al. [53] as a relevant model for NF1; these models account for the internal, external, and neurobiological factors that influence cognitive functions and how those interconnected cognitive functions (e.g., attention/executive functions, communication, socio-emotional factors) contribute to the social deficits observed in individuals with RASopathies.

### Limitations

The selection of study measures to examine detailed aspects of social behavior, and particularly empathetic and prosocial behavior, was limited by the small number of available measures with normative data. Multi-method and multi-informant approaches that combine parent- and teacher-report measures with direct observation of child behavior could provide additional valuable information regarding social behavior across settings, although such methods are challenging to employ in investigations of rare populations. Additional factors such as home or school environment, family socioeconomic status, family characteristics (e.g., parent diagnosis of RASopathy), and use of medication or behavioral treatment approaches may also have influenced social behavior ratings but were not measured in this study. Differences in the relative reliance on particular recruitment sources (conferences versus online research networks) may also have led to demographic differences among the RASopathy and iASD groups. Finally, use of electronic survey methodology, while helpful to facilitate recruitment of a larger cohort, precluded the observation of parents/caregivers during survey completion and the ability to provide clarification regarding any questions that arose.

## Conclusions

While social function is a well-established area of concern for many children with RASopathies, this study provides evidence that a relative strength in empathetic behavior is frequently observed, thus differentiating the social functioning problems experienced by these children from those experienced by children with iASD. Furthermore, the patterns of psychological factors associated with different areas of social strength and weakness differed among children with RASopathy and children with iASD. For children with RASopathies, problems with social competence were primarily associated with hyperactivity/inattention and emotional difficulties, while problems with empathy were most related to communication challenges and hyperactivity/inattention. This pattern contrasted to social behavior problems for children with iASD, which were predominately associated with communication problems. Thus, it is recommended that interventions for social difficulties target the specific social difficulties experienced by that individual as well as the developmental and neuropsychological factors underlying those difficulties.

## Supplementary Information

**Additional file 1 Table S1**. Description of the social behavior survey subscales. **Table S2**. Child demographics for verbal participants rated using social behavior surveys. **Table S3**. Percentage of each group showing clinically significant impairment (> 2 SD) on the social behavior scales. **Table S4**. SDQ Scores Among RASopathy and Idiopathic ASD Groups. **Table S5**. Scores on social behavior measures for children with comorbid RASopathy+ASD as compared to idiopathic ASD

## Data Availability

The data that support the findings of this study are available from the authors upon reasonable request.

## Abbreviations

ADHD: Attention deficit hyperactivity disorder
ASD: Autism spectrum disorder
iASD: Idiopathic ASD
RAS+ASD: RASopathy with co-occurring ASD diagnosis
SDQ: Strengths and Difficulties Questionnaire
SEARS: Social Emotional Assets and Resilience Scales
