# Supplementary material for: Social behavior in RASopathies and idiopathic autism
Source: J Neurodev Disord. 2022 Jan 12;14:5. doi: 10.1186/s11689-021-09414-w (PMC8753327; doi:10.1186/s11689-021-09414-w)
Supplement: Supplementary file 1 — Additional file 1 Table S1. Description of the social behavior survey subscales. Table S2. Child demographics for verbal participants rated using social behavior surveys. Table S3. Percentage of each group showing clinically significant impairment (> 2 SD) on the social behavior scales. Table S4. SDQ Scores Among RASopathy and Idiopathic ASD Groups. Table S5. Scores on social behavior measures for children with comorbid RASopathy+ASD as compared to idiopathic ASD [file 11689_2021_9414_MOESM1_ESM.docx]

S1. Description of the social behavior survey subscales

| Scale | Description |
| --- | --- |
| SEARS |  |
| Social Competence | This scale measures the child’s “ability to maintain friendships with peers, engage in effective verbal communication, and feel comfortable around groups of peers” (65). Items reflect the child’s likability, friendships, social communication skills, and leadership skills. |
| Empathy | This scale measures the child’s “ability to understand and relate to others’ situations and feelings” (65). Items assess the child’s frequency of helping behaviors, empathic behaviors, and demonstrated insight into the emotional experiences of others. |
| SDQ |  |
| Emotional Symptoms | This scale assesses the frequency of worrying and anxiety, mood problems, and physical symptoms that may indicate anxiety or mood disturbance. |
| Conduct Problems | This scale assesses the frequency of dishonest or disobedient behavior, fighting, and anger or disruptive behaviors. |
| Hyperactivity/Inattention | This scale assesses the frequency of symptoms of hyperactivity and inattention. Two items focus on restless behaviors, two assesses inattention, and one assesses impulsivity. |
| Peer Relationship Problems | These items measure the child’s likability, ability to get on with adults versus peers, friendships, solitariness, and frequency of being bullied. |
| Prosocial Behavior | This scale assesses helping and sharing behaviors, treatment of younger children, and empathy. |

S2. Child demographics for verbal participants rated using social behavior surveys

|  | NF1  (n =70) | NS  (n =70) | CFCS  (n =25) | CS  (n =15) | RASopathy  (n =180) | iASD  (n =97) |
| --- | --- | --- | --- | --- | --- | --- |
| Participant demographics |  |  |  |  |  |  |
| Age of Child (M (SD)) | 9.9 (4.4) | 9.4 (4.2) | 9.9 (4.3) | 11.5 (4.4) | 9.8 (4.3) | 10.5 (3.7) |
| Gender (N (% Male)) | 36 (51) | 34 (49) | 8 (32) | 8 (53) | 86 (48) | 72 (74) |
| Race (N (%)) |  |  |  |  |  |  |
| American Indian or Alaska Native | 0 (0) | 1 (1) | 1 (4) | 0 (0) | 2 (1) | 3 (3) |
| Asian | 2 (3) | 4 (6) | 1 (4) | 2 (13) | 9 (5) | 3 (3) |
| Black or African  American | 4 (6) | 3 (4) | 0 (0) | 0 (0) | 7 (4) | 5 (5) |
| Native Hawaiian or Other Pacific Islander | 1 (1) | 1 (1) | 0 (0) | 2 (13) | 4 (2) | 1 (1) |
| White | 64 (92) | 66 (94) | 24 (96) | 14 (93) | 168 (93) | 88 (91) |
| Other | 1 (1) | 1 (1) | 2 (8) | 1 (7) | 5 (3) | 6 (6) |
| Ethnicity |  |  |  |  |  |  |
| Hispanic/Latinx | 13 (19) | 4 (6) | 1 (4) | 1 (7) | 19 (11) | 15 (16) |
| Medical Complications |  |  |  |  |  |  |
| Preterm birth | 5 (7) | 11 (16) | 6 (24) | 6 (40) | 28 (16) | 9 (9) |
| Seizures | 9 (13) | 7 (10) | 10 (40) | 2 (13) | 28 (16) | 1 (1) |
| Tumor | 30 (43) | 4 (6) | 0 (0) | 0 (0) | 34 (19) | 0 (0) |
| Visual Impairment | 19 (28) | 36 (51) | 17 (68) | 10 (67) | 82 (46) | 20 (21) |
| Hearing Impairment | 3 (4) | 15 (21) | 3 (12) | 0 (0) | 21 (12) | 2 (2) |

S3. Percentage of each group showing clinically significant impairment (>2 SD) on the social behavior scales

|  | **SEARS Scale** | | **SDQ Scale** | | | | |
| --- | --- | --- | --- | --- | --- | --- | --- |
| **Group** | **Social Competence** | **Empathy** | **Emotional Symptoms** | **Conduct problems** | **Hyperactivity/Inattention** | **Peer Problems** | **Lack of Prosocial Behavior** |
| **CFCS (N=25)** | 24 | 14 | 24 | 4 | 40 | 36 | 44 |
| **CS (N=15)** | 0 | 0 | 20 | 0 | 20 | 33 | 20 |
| **NF1 (N=70)** | 23 | 7 | 34 | 11 | 33 | 43 | 17 |
| **NS (N=70)** | 17 | 9 | 27 | 14 | 31 | 34 | 20 |
| **iASD (N=97)** | 48 | 40 | 29 | 17 | 46 | 58 | 44 |

| S4. SDQ Scores Among RASopathy and Idiopathic ASD Groups | | | | | | | |
| --- | --- | --- | --- | --- | --- | --- | --- |
|  |  | **SDQ (*z*-Score)** | | | | | |
| **Group** |  | **Emotional Problems** | **Conduct problems** | **Hyperactivity** | **Peer Problems** | **Lack of Prosocial Behavior*** | **Total Problems** |
| **CFCS (N=25)** | Mean | 1.15 | 0.31 | 1.80 | 1.48 | 1.70 | 1.68 |
|  | (SD) | (1.53) | (0.97) | (1.04) | (1.41) | (1.35) | (1.05) |
| **CS (N=15)** | Mean | 1.24 | 0.19 | 1.07 | 1.22 | 0.96 | 1.26 |
|  | (SD) | (1.10) | (0.89) | (0.99) | (1.25) | (1.03) | (1.11) |
| **NF1 (N=70)** | Mean | 1.35 | 0.49 | 1.31 | 1.36 | 0.62 | 1.52 |
|  | (SD) | (1.54) | (1.12) | (1.11) | (1.42) | (1.04) | (1.28) |
| **NS (N=70)** | Mean | 1.13 | 0.61 | 1.23 | 1.29 | 0.71 | 1.44 |
|  | (SD) | (1.40) | (1.28) | (1.23) | (1.49) | (1.32) | (1.36) |
| **Idiopathic ASD (N=97)** | Mean | 1.32 | 0.71 | 1.59 | 2.21 | 1.79 | 1.91 |
|  | (SD) | (1.29) | (1.10) | (1.03) | (1.11) | (1.24) | (1.03) |

*The Prosocial scale of the SDQ was reverse-scored here to match the directionality of the other four SDQ scales, where higher z-scores indicate greater problems

| S5. Scores on social behavior measures for children with comorbid RASopathy+ASD as compared to idiopathic ASD | | | | | | | | | | |  |
| --- | --- | --- | --- | --- | --- | --- | --- | --- | --- | --- | --- |
|  | **RASopathy + ASD** | | | | **Idiopathic ASD** | | | |  | |  |
| **Measure** | n | Mean | (SD) | % Clinical Impairment (≥2 SD below mean) | n | Mean | (SD) | % Clinical Impairment (≥2 SD below mean) | Mean Difference (95% CI) | |  |
| **Social Emotional Assets & Resilience Scales (SEARS)** | | | | | | | | | | |  |
| **Social Competence** | 20 | 30.65 | (8.29) | 45 | 87 | 30.49 | (7.64) | 48 | .16 | -3.66 to 3.97 |  |
| **Empathy** | 20 | 35.60 | (8.56) | 25 | 88 | 32.99 | (9.39) | 40 | 2.61 | -1.93 to 7.15 |  |
|  | n | Mean | (SD) | % Clinical Impairment (≥2 SD above mean) | n | Mean | (SD) | % Clinical Impairment (≥2 SD above mean) | Mean Difference (95% CI) | |  |
| **Strengths & Difficulties Questionnaire (SDQ)** | | | | | | | | | | |  |
| **Emotional Symptoms** | 22 | 1.88 | (1.65) | 41 | 97 | 1.32 | (1.29) | 29 | .56 | -.08 to 1.20 |  |
| **Conduct Problems** | 22 | 0.66 | (0.91) | 9 | 97 | 0.71 | (1.10) | 17 | -.04 | -.54 to .45 |  |
| **Hyperactivity/Inattention** | 22 | 1.86 | (1.00) | 50 | 97 | 1.59 | (1.03) | 46 | .27 | -.21 to .75 |  |
| **Peer Relationship Problems** | 22 | 1.80 | (1.34) | 55 | 97 | 2.21 | (1.11) | 58 | -.41 | -.95 to .13 |  |
| **Prosocial Behavior** | 22 | 1.53 | (0.96) | 36 | 97 | 1.79 | (1.24) | 44 | -.26 | -.83 to .30 |  |
| SEARS scores are reported in T-scores with a mean of 50 and a standard deviation of 10. Higher SEARS scores indicate fewer problems; SDQ scores are reported in z-scores with a mean of 0 and a standard deviation of 1. Higher SDQ scores indicate more problems. | | | | | | | | | | |  |
